# Means of Averting Death from Chloroform

**Published:** 1865-12

**Authors:** 


### MEANS OF AVERTING DEATH FROM CHLOROFORM.

---

Dr. J. Bullar, of Southampton, states that a plan has been successfully practised in the hospital of that place, for reviving those who are sinking from the administration of chloroform. This plan consists in the assistants at the operation striking the patient with the flat hand in the most vigorous and rapid way on all exposed parts of the skin (the trunk, the legs, the arms, and the face), and to continue this until the pulse and breathing return. The mouth is also kept open and the tongue drawn forward.

In one case, which Dr. Bullar briefly notices, he says: — "It is difficult to estimate the time, but certainly from five to ten minutes elapsed before the heart acted, and not until both legs were blue with ecchymoses. Had not the staff of the hospital had faith in this remedy, the case at one time seemed so hopeless that other plans might have been attempted and time lost; but this faith is necessary for perseverance. Every part of the skin should be rapidly exposed, and every one should assist and strike with his flat hand as sharply and rapidly as he can, and continue it.

In this instance the immediate local effect in filling the parts of the skin which were struck and around them with red blood whilst the heart and breathing had ceased, shows that this plan is a stimulus of the reflex nervous function of a very vigorous kind, which can be immediately applied to the cutaneous ends of the nerves over a large surface without any apparatus, and with the greatest ease.

As the sympathetic nerve presides over the capillaries, and over the heart, the excitement in both by this means is readily explained by the direct communications of the ganglia of the sympathetic with the roots of the spinal nerves. According to the doctrine of the correlation of forces, the mechanical force of the hands of the strikers is converted into the nerve-force of the patient, and is conveyed by the sensitive nerves of the skin to the spinal cord, and is reflected from it, through the sympathetic, to the heart and capillaries.—*Amer. Jour. of Med. Science*, from *Med. Times and Gazette.*

